# Supplementary material for: Expressional artifact caused by a co-injection marker rol-6 in C. elegans
Source: PLoS One. 2019 Dec 4;14(12):e0224533. doi: 10.1371/journal.pone.0224533 (PMC6892501; doi:10.1371/journal.pone.0224533)
Supplement: S1 Table — (DOCX) [file pone.0224533.s001.docx]

**S1 Table. Promoter::GFP test genes and the fragments assayed for CP09 expression**

| Strain name | Transgene | Amplified region (bp) | Reporter for |
| --- | --- | --- | --- |
| EM1206 | *bxEx171[C29F5.3p::gfp+rol-6(su1006)];him-5(e1490)V* | -1102 ~ -4 | *C29F5.3* |
| EM1207 | *bxEx172[snt-3p::gfp+rol-6(su1006)];him-5(e1490)V* | -2259 ~ -6 | *snt-3* |
| EM1208 | *bxEx173[dpf-1p::gfp+rol-6(su1006)];him-5(e1490)V* | -2058 ~ -6 | *dpf-1* |
| EM1209 | *bxEx174[sul-2p::gfp+rol-6(su1006)];him-5(e1490)V* | -2021 ~ -4 | *sul-2* |
| EM1210 | *bxEx175[DH11.5p::gfp+rol-6(su1006)];him-5(e1490)V* | -2005 ~ -1 | *DH11.5* |
| EM1211 | *bxEx176[F27C1.11p::gfp+rol-6(su1006)];him-5(e1490)V* | -893 ~ -1 | *F27C1.11* |
| EM1212 | *bxEx177[M117.1p::gfp+rol-6(su1006)];him-5(e1490)V* | -2096 ~ -1 | *M117.1* |
| EM1213 | *bxEx178[T19A6.4p::gfp+rol-6(su1006)];him-5(e1490)V* | -999 ~ -1 | *T19A6.4* |
| EM1214 | *bxEx179[W10C8.5p::gfp+rol-6(su1006)];him-5(e1490)V* | -2065 ~ -1 | *W10C8.5* |
| - | *bxEx180[cka-2p::gfp+rol-6(su1006)];him-5(e1490)V* | -2049 ~ -1 | *cka-2* |

Original transgenes were previously described in [9].
