# Supplementary material for: Expressional artifact caused by a co-injection marker rol-6 in C. elegans
Source: PLoS One. 2019 Dec 4;14(12):e0224533. doi: 10.1371/journal.pone.0224533 (PMC6892501; doi:10.1371/journal.pone.0224533)
Supplement: S1 Text — (DOCX) [file pone.0224533.s002.docx]

**S1 Fig. pRF4 sequence (7,271 bp)**

*rol-6(su1006)*

su1006 mutation

Minimum homology with GFP constructs

GAATTCttatcatcttcggttttgataaaattgtggtgtagtccataatgaaataaaattagaaacaattgaaaatgagaataattggaaggaaagagaaccggtcatttagaatgaacacaatagcttttttagtgattgttgcgtaaattttatgacattacgtcattgaatcctgaagaagacaatgtttaaatgttctccagatggaattaaatgtataattaagctcgagatttttatccgagtttttataaaagttggtgagacccaggattaatcaaaaacaagtattgacagttccgtcattcataactccaccgactgctcaagttggcaaaagtcaacttttaaagtgggttataaagagacttcgatttttcagatttgcaaatcatttgaaagcatttgttaaactagatataaatacatagttttcctcaatcaggaaaatcaaaccactgatcatctgatcagctgtcttctaacaggatgcactattaatgaattaaacgaactcggtctattcactcggataattgcaacaatgttagttctatttgatttatttcggcgcgatccgtagcgacactttcttccagcaaccatgtgatgctgatttctatcatccgaagagagaaggaaacatggagaggagcggttcaagaacaggtgaatctgaacctccttttccgtgtgtgtggaaatatacacaaacaagtgactgatgaaggcgggaaaaagaaaaacgatggattgagttatctgggtgaagaatcttagacaaagagtttgcagaaaaagcttgttcttggaaaaataaaaaactagacaatttttaaaaaggaaaactacttatttatcacttcaaaaccagaacccttttgacaatctttaactgtttgaacttcgtattagatctcagcagcaaaataatagttgaaaaaattttataagaaaagttttcgtgttcagttgaaaacatccaaaacagaaagtatttttatttactgataattccttaatacacatctattaccgtatttggggcttctaaaaatatattctagtcgtttcaaagttcaagcacattacgttaattcatagatcaaatccactaaacctcaaccatcactttttctaatttcttccacttcaaaaaaaaactaatcatcgtggcttcttcccgttccatgtcatatggtcaacattaatcagtcgatggaattggcctttttaccgaaaatacgaagattccacgcatcattgcaacattttcgtcataacctcgctagcccaaatcggttgtaggatccccgaatcgcattgtggttgagcgtatgtccttcaaaaagtccaacaaaaactcgaataccgtttgccatcggatggtgttgttgaagagttttaactataagctacgcccatccggacgtacgcgagaagccctttctgttgaatgggtgggcggcggaagaaaaaacttcttaacacggcttttcggtttttctaccctatatattggaggataggtgaaggatattttggagaatatacgattagcggcaatcacaatgacatccttatgtatatgcattctttttattgtttcctgatatttattctctctttatcatttcactttttcttggcactcgtccaaaaatctcaattaaaaatttctggatatatctttagatctaactgaaaatttccagATGACCCTAACTACGGCGACGTCCGGCGCCATTGTATTTTCTGGAGCCACTTTGTTGGTTTCTCTTTTTGCCGCTGCTTCGCTTTACAGTCAAGTTTCCAATATCTGGAATGAGCTGGATGCCGAAATCGCCAACTTCAGAgtgagtttcctatatagcatttctaattcactgataatttcaattatttagAGTCTCACTGAAGACATGTGGGTTGATATGGTTAAACTTGGAGCAGGAACCGCTTCCAACCGTGTGAGATGTCAACAATATGGAGGATATGGAGCCACTGGTGTTCAGCCACCAGCACCAACTCCAAACCCATATGGAGGATATGGAGCAAGCCAGCCAGCTCCACCAGAGAAATTCCCAGATGGTATACCAAATGGAGGAAATCAACCAAAGTTCCCAGGAGGTGGATTCCCAGATGGTCCATTCCCGAATGGAGGAGGACCACGTGGAGGAAATCAATGTCAATGCACTGTTGAGAACTCATGCCCACCAGGACCAGCTGGACCAGAAGGAGAGGAAGGACCAGATGGGCACGATGGACAAGACGGAGTCCCAGGATTTGACGGAAAAGATGCCGAAGATGTTCAAAACACCCCACCAACAGGATGCTTCACCTGTCCACAAGGACCACTTGGACCACAAGGACCAAATGGAGCTCCAGGACTCAGAGGAATGCGCGGAGCTCGTGGACAACCAGGACGTCCAGGAAGAGACGGAAACCCAGGAATGCCAGGAGACTGTGGACCACCAGGGGCCCCAGGATCCGATGGAAAGCCAGGATCTCCAGGAGGAAAGGGAGACGATGGAGAGAGACCATTGGGCCGCCCAGGACCAAGAGGACCACCAGGAGAGGCTGGGCCAGAAGGACCACAGGGACCAACTGGAAGAGATGCTTATCCAGGACAGTCTGGACCACAAGGAGAGCCAGGCCTTCAAGGATATGGAGGAGCTGCTGGAGAGGACGGgtaggatgaaaaaaatattaaacgacttcttgtaaatataaaaatttcagACCAGAAGGACCACCAGGAGCCCCAGGACTTCCAGGAAAAGATGCCGAATACTGCAAATGCCCAGGAAGAGAAGGAGATGCTGGACGAAGTGCCAGACGTCATCGCAAATTCCAATTGTAGacaaattcatgacatttttccaaaaaataaaacatacttctcaaaattttttgttttgtatttggtttaccatggatgttaagaactttcttggggaaaataattaaaatacaaaactgtataaattatgtcacaagcttggaagtgcatttgcaaaaaaacactggtgtagaagaacaagaggttagtatgacttgaacagaataactaatagaaattggagagcatccttttagaaggaaaaatgcacgacctagaacagctattaatctgtatttggcactcgatttatattgctagaaatggaacctattagtataaggaaaaataagagaaaaggtgagaaggtcggaaaaaacacttcatgaatatatttagacgaggaaaaaacgaaggaaataatgggagagtgacgatagaaagaaggaaaacgttgaaaactgagtcgaaaagaaagaaaagaacgaaggtgaacaaaaacgacaaagttttgaaaatcatttcaattttcagatttcagttcaagaccgagtagttggaaaacatgtgacctcaaaaaaaaaaacaaaaaatttcacttaaaggggaaagaagtgaagtagaacttctgcataatcatcaaaagattcccgaatagcttggttccaaaagttcaaacacaaattacttttggaaactttcatttgttttgaactgtagcggcatgtcgatttttttcaaaatacaaattacaatagaaaatcaggaaaacaattggaagtgattttggaaaaaaaaacgttgaggagatttgagcacgagacaaacaatttctgtaaatttcagttcaaaagcttgaaaacggttctttcttgcaaacaaaacctagtacctcttcactttaacatacacttcacgaacaagttttcttttgaaaataatttctgaaaatcacgttaacgaatctcaaaattcaggaaatgctcaaaaatgtctatttgcgtactacgaaattcccgaaaaaacatctgtgacttttttctctctttttgttcgaaaaaataacttatgtcgccgtggtggtgccctcgggaatcggaaagcgtcaatgttgtgtttgttgttgttatcccaaacgagaagatgaataagaggaggaaagatgaacggaaagcgagaaaaaaagaagatggaacacgtacatgagcttctcctgctttgaatacGAATTCgccctatagtgagtcgtattacaattcactggccgtcgttttacaacgtcgtgactgggaaaaccctggcgttacccaacttaatcgccttgcagcacatccccctttcgccagctggcgtaatagcgaagaggcccgcaccgatcgcccttcccaacagttgcgcagcctgaatggcgaatggacgcGCCCTGTAGCGGCGCATTAAGCGCGGCGGGTGTGGTGGTTACGCGCAGCGTGACCGCTACACTTGCCAGCGCCCTAGCGCCCGCTCCTTTCGCTTTCTTCCCTTCCTTTCTCGCCACGTTCGCCGGCTTTCCCCGTCAAGCTCTAAATCGGGGGCTCCCTTTAGGGTTCCGATTTAGTGCTTTACGGCACCTCGACCCCAAAAAACTTGATTAGGGTGATGGTTCACGTAGTGGGCCATCGCCCTGATAGACGGTTTTTCGCCCTTTGACGTTGGAGTCCACGTTCTTTAATAGTGGACTCTTGTTCCAAACTGGAACAACACTCAACCCTATCTCGGTCTATTCTTTTGATTTATAAGGGATTTTGCCGATTTCGGCCTATTGGTTAAAAAATGAGCTGATTTAACAAAAATTTAACGCGAATTTTAACAAAATATTAACGCTTACAATTTCctgatgcggtattttctccttacgcatctgtgcggtatttcacaccgcatatggtgcactctcagtacaatctgctctgatgccgcatagttaagccagccccgacacccgccaacacccgctgacgcgccctgacgggcttgtctgctcccggcatccgcttacagacaagctgtgaccgtctccgggagctgcatgtgtcagaggttttcaccgtcatcaccgaaacgcgcgagacgaaagggcctcgtgatacgcctatttttataggttaatgtcatgataataatggtttcttagacgtcaggtggcacttttcggggaaatgtgcgcggaacccctatttgtttatttttctaaatacattcaaatatgtatccgctcatgagacaataaccctgataaatgcttcaataatattgaaaaaggaagagtatgagtattcaacatttccgtgtcgcccttattcccttttttgcggcattttgccttcctgtttttgctcacccagaaacgctggtgaaagtaaaagatgctgaagatcagttgggtgcacgagtgggttacatcgaactggatctcaacagcggtaagatccttgagagttttcgccccgaagaacgttttccaatgatgagcacttttaaagttctgctatgtggcgcggtattatcccgtattgacgccgggcaagagcaactcggtcgccgcatacactattctcagaatgacttggttgagtactcaccagtcacagaaaagcatcttacggatggcatgacagtaagagaattatgcagtgctgccataaccatgagtgataacactgcggccaacttacttctgacaacgatcggaggaccgaaggagctaaccgcttttttgcacaacatgggggatcatgtaactcgccttgatcgttgggaaccggagctgaatgaagccataccaaacgacgagcgtgacaccacgatgcctgtagcaatggcaacaacgttgcgcaaactattaactggcgaactacttactctagcttcccggcaacaattaatagactggatggaggcggataaagttgcaggaccacttctgcgctcggcccttccggctggctggtttattgctgataaatctggagccggtgagcgtgggtctcgcggtatcattgcagcactggggccagatggtaagccctcccgtatcgtagttatctacacgacggggagtcaggcaactatggatgaacgaaatagacagatcgctgagataggtgcctcactgattaagcattggtaactgtcagaccaagtttactcatatatactttagattgatttaaaacttcatttttaatttaaaaggatctaggtgaagatcctttttgataatctcatgaccaaaatcccttaacgtgagttttcgttccactgagcgtcagaccccgtagaaaagatcaaaggatcttcttgagatcctttttttctgcgcgtaatctgctgcttgcaaacaaaaaaaccaccgctaccagcggtggtttgtttgccggatcaagagctaccaactctttttccgaaggtaactggcttcagcagagcgcagataccaaatactgttcttctagtgtagccgtagttaggccaccacttcaagaactctgtagcaccgcctacatacctcgctctgctaatcctgttaccagtggctgctgccagtggcgataagtcgtgtcttaccgggttggactcaagacgatagttaccggataaggcgcagcggtcgggctgaacggggggttcgtgcacacagcccagcttggagcgaacgacctacaccgaactgagatacctacagcgtgagctatgagaaagcgccacgcttcccgaagggagaaaggcggacaggtatccggtaagcggcagggtcggaacaggagagcgcacgagggagcttccagggggaaacgcctggtatctttatagtcctgtcgggtttcgccacctctgacttgagcgtcgatttttgtgatgctcgtcaggggggcggagcctatggaaaaacgccagcaacgcggcctttttacggttcctggccttttgctggccttttgctcacatgttctttcctgcgttatcccctgattctgtggataaccgtattaccgcctttgagtgagctgataccgctcgccgcagccgaacgaccgagcgcagcgagtcagtgagcgaggaagcggaagagcgcccaatacgcaaaccgcctctccccgcgcgttggccgattcattaatgcagctggcacgacaggtttcccgactggaaagcgggcagtgagcgcaacgcaattaatgtgagttagctcactcattaggcaccccaggctttacactttatgcttccggctcgtatgttgtgtggaattgtgagcggataacaatttcacacaggaaacagctatgaccatgattacgccaagctcgaaattaaccctcactaaagggaacaaaagcttgcatgcctgcaggtcgactctagaggatccccgggtaccgagctc
