# Supplementary material for: Expressional artifact caused by a co-injection marker rol-6 in C. elegans
Source: PLoS One. 2019 Dec 4;14(12):e0224533. doi: 10.1371/journal.pone.0224533 (PMC6892501; doi:10.1371/journal.pone.0224533)
Supplement: Attachment — Submitted filename: Rebuttal letter.docx [file pone.0224533.s003.docx]

**Jin et al.**

**RESPONSE TO REVIEWERS**

**Journal Requirements:**

1. When submitting your revision, we need you to address these additional requirements.

Please ensure that your manuscript meets PLOS ONE's style requirements, including those for file naming. The PLOS ONE style templates can be found at

http://www.journals.plos.org/plosone/s/file?id=wjVg/PLOSOne_formatting_sample_main_body.pdf and http://www.journals.plos.org/plosone/s/file?id=ba62/PLOSOne_formatting_sample_title_authors_affiliations.pdf

We have revised the text to meet the style requirements.

2.  We note that Figure(s) [1A] in your submission contain copyrighted images. All PLOS content is published under the Creative Commons Attribution License (CC BY 4.0), which means that the manuscript, images, and Supporting Information files will be freely available online, and any third party is permitted to access, download, copy, distribute, and use these materials in any way, even commercially, with proper attribution. For more information, see our copyright guidelines: http://journals.plos.org/plosone/s/licenses-and-copyright.

We require you to either (1) present written permission from the copyright holder to publish these figures specifically under the CC BY 4.0 license, or (2) remove the figures from your submission:

1.         You may seek permission from the original copyright holder of Figure(s) [1A] to publish the content specifically under the CC BY 4.0 license.

We recommend that you contact the original copyright holder with the Content Permission Form (http://journals.plos.org/plosone/s/file?id=7c09/content-permission-form.pdf) and the following text:

“I request permission for the open-access journal PLOS ONE to publish XXX under the Creative Commons Attribution License (CCAL) CC BY 4.0 (http://creativecommons.org/licenses/by/4.0/). Please be aware that this license allows unrestricted use and distribution, even commercially, by third parties. Please reply and provide explicit written permission to publish XXX under a CC BY license and complete the attached form.”

Please upload the completed Content Permission Form or other proof of granted permissions as an "Other" file with your submission.

In the figure caption of the copyrighted figure, please include the following text: “Reprinted from [ref] under a CC BY license, with permission from [name of publisher], original copyright [original copyright year].”

We have uploaded the completed Content Permission Form. Also, we have added the text in the figure caption for copyright information.

3. We note that you have included the phrase “data not shown” in your manuscript. Unfortunately, this does not meet our data sharing requirements. PLOS does not permit references to inaccessible data. We require that authors provide all relevant data within the paper, Supporting Information files, or in an acceptable, public repository. Please add a citation to support this phrase or upload the data that corresponds with these findings to a stable repository (such as Figshare or Dryad) and provide and URLs, DOIs, or accession numbers that may be used to access these data. Or, if the data are not a core part of the research being presented in your study, we ask that you remove the phrase that refers to these data.

As the result was a none-or-all event and negative in a test, we think it can be shown as a simple description without mentioning about data availability. Thus, we have removed the phrase “data not shown”.

**Review Comments to the Author**

Reviewer #1: This manuscript describes a relatively minor finding, but that finding is described in a concise manner. Furthermore, the implications for others working on C. elegans male tail biology are significant and this justifies publication as the information should be in the public domain. It is important to be aware of the presence of a cell specific enhancer in a common transformation marker as this could affect conclusions drawn from experiments.

The manuscript is very well put together. A few minor issues are identified below.

Line 51. Information on the original genes and the fragments assayed ought to be included perhaps in a supplementary table.

The original information was described in Experimental Procedures section in our previous paper. To avoid repetition and clearly inform the source of information, we have indicated the source in the text:

“for information on the genes and the promoter fragments assayed, see Experimental Procedures section in [ref]”.

Line 94 – 101. Some clarification of the cloning used to identify what part of pRF4 is responsible for driving expression in CP09 is needed in the Results and Discussion text, in addition to that provided in the Materials and Methods section. It is not clear from the general description of sub-cloning of pRF4, if the minimum gfp homology region is in the vector or insert fragment.

We agree that our description about the minimum gfp homology region was not clear. The minimum homology region is located upstream of the GFP coding region of pPD95.75. In the sub-cloning procedure, the minimum homology region was excluded so that both the vector and rol-6 fragments did not contain the minimum homology sequence.

To clarify this, we added the following text in Results and Discussion section:

“The minimum homology sequence is located upstream of the GFP coding region of pPD95.75 and included in most promoter::GFP fusion constructs, as this inclusion ensures that an artificial intron is placed in front of the GFP sequence for efficient reporter gene expression [ref].”

“…we divided the pRF4 plasmid except for the minimum homology region into two fragments,…”

Also, cloning of the pRF4 vector fragment into pPD95.77, would lead to a repeat of the pUC vector parts in the same plasmid which would be expected to be unstable in E. coli and I would be surprised if this really was achieved.

We re-checked the sub-cloning of the vector fragment::GFP and found no errors in making it. This construct may be unstable due to the repetition of vector sequences, but we did not find any difficulties to sub-clone and amplify this construct using E. coli.

Methods. Restriction enzyme names should include italics.

We have revised the text accordingly.

References. Gene and species names should be italicised.

We have revised the text accordingly.

Figs. 1 and 2. Reference is to “CP09” throughout the paper apart from in Figs 1 and 2 where it is “CP9”.

We have revised the figures accordingly.

Fig. 3. Title for panel C is in square brackets and is not needed.

We have removed the title for panel C in Figure 3.
