# Supplementary material for: Expressional artifact caused by a co-injection marker rol-6 in C. elegans
Source: PLoS One. 2019 Dec 4;14(12):e0224533. doi: 10.1371/journal.pone.0224533 (PMC6892501; doi:10.1371/journal.pone.0224533)
Supplement: Attachment — Submitted filename: Rebuttal letter_v2.docx [file pone.0224533.s004.docx]

**Jin et al.**

**RESPONSE TO REVIEWERS**

Thank you for submitting your revised manuscript to PLOS ONE. This revised version of the manuscript addresses most the points raised during the initial review process. However, it would be preferable if you included the methodological information referenced within this article rather than as a call back :

“for information on the genes and the promoter fragments assayed, see Experimental Procedures section in [ref]To avoid repetition and clearly inform the source of information, we have indicated the source in the text: 
“for information on the genes and the promoter fragments assayed, see Experimental Procedures section in [ref]

This would allow readers to access all the pertinent information without juggling between two references.

We have added S1 Table to indicate information on the genes and the promoter fragments assayed.
